# Supplementary material for: A single-cell transcriptomic atlas tracking the neural basis of division of labour in an ant superorganism
Source: Nat Ecol Evol. 2022 Jun 16;6(8):1191–204. doi: 10.1038/s41559-022-01784-1 (PMC9349048; doi:10.1038/s41559-022-01784-1)
Supplement: Supplementary file 1 — Reporting Summary [file 41559_2022_1784_MOESM1_ESM.pdf]

nature portfolio

Corresponding author(s): Chuanyu Liu, Guojie Zhang, Weiwei Liu

Last updated by author(s): Apr 21, 2022

Reporting Summary

Nature Portfolio wishes to improve the reproducibility of the work that we publish. This form provides structure for consistency and transparency in reporting. For further information on Nature Portfolio policies, see our Editorial Policies and the Editorial Policy Checklist.

Statistics

For all statistical analyses, confirm that the following items are present in the figure legend, table legend, main text, or Methods section.

n/a	Confirmed	
☐	☒	The exact sample size (*n*) for each experimental group/condition, given as a discrete number and unit of measurement
☒	☐	A statement on whether measurements were taken from distinct samples or whether the same sample was measured repeatedly
☐	☒	The statistical test(s) used AND whether they are one- or two-sided<br>*Only common tests should be described solely by name; describe more complex techniques in the Methods section.*
☒	☐	A description of all covariates tested
☐	☒	A description of any assumptions or corrections, such as tests of normality and adjustment for multiple comparisons
☐	☒	A full description of the statistical parameters including central tendency (e.g. means) or other basic estimates (e.g. regression coefficient) AND variation (e.g. standard deviation) or associated estimates of uncertainty (e.g. confidence intervals)
☐	☒	For null hypothesis testing, the test statistic (e.g. *F*, *t*, *r*) with confidence intervals, effect sizes, degrees of freedom and *P* value noted<br>*Give P values as exact values whenever suitable.*
☒	☐	For Bayesian analysis, information on the choice of priors and Markov chain Monte Carlo settings
☒	☐	For hierarchical and complex designs, identification of the appropriate level for tests and full reporting of outcomes
☐	☒	Estimates of effect sizes (e.g. Cohen's *d*, Pearson's *r*), indicating how they were calculated

Our web collection on statistics for biologists contains articles on many of the points above.

Software and code

Policy information about availability of computer code

Data collection	No software was used.
Data analysis	Softwares used to analyze the data were described in details in the Methods section of the manuscript and listed below:<br>Drop-seq_tools-1.13, STAR (2.6.1a_08-27), Seurat (v3.1.5), SOAPnuke (v 2.1.0), Hisat2 (v 2.1.0),<br>DESeq2 (v1.22.0), BLASTP (blast-2.2.26), MetaNeighbor, Cytoscape (v 3.8.2), scCODA (v 0.1.6), AMIRA (v6.4), EZ-MET (x64, 6.0.7543), R (v.3.6) and R (v4.0.5).

For manuscripts utilizing custom algorithms or software that are central to the research but not yet described in published literature, software must be made available to editors and reviewers. We strongly encourage code deposition in a community repository (e.g. GitHub). See the Nature Portfolio guidelines for submitting code & software for further information.

Data

Policy information about availability of data

All manuscripts must include a data availability statement. This statement should provide the following information, where applicable:

- Accession codes, unique identifiers, or web links for publicly available datasets
- A description of any restrictions on data availability
- For clinical datasets or third party data, please ensure that the statement adheres to our policy

The raw snRNA-seq data of M. pharaonis generated in this study are deposited in the CNGB Nucleotide Sequence Archive (CNSA) with accession number CNP0001472. The reference genome, gene models, functional annotations of protein-coding genes, full marker gene list of each cell cluster, and all in-house scripts are deposited in the figshare repository under the link https://doi.org/10.6084/m9.figshare.16616353.

Field-specific reporting

Please select the one below that is the best fit for your research. If you are not sure, read the appropriate sections before making your selection.

☒ Life sciences ☐ Behavioural & social sciences ☐ Ecological, evolutionary & environmental sciences

For a reference copy of the document with all sections, see nature.com/documents/nr-reporting-summary-flat.pdf

Life sciences study design

All studies must disclose on these points even when the disclosure is negative.

Sample size	We set 4 to 5 replicates for each of the four adult phenotypes of Monomorium pharaonis (i.e. workers, queens, gynes or males), as this number of replicate is suitable for controlling variation resulting from different bathes of library construction or sequencing. We obtained a total of 206,367 high-quality nuclei from the four adult phenotypes. This is 1.3 to 4 times the estimated cell number of 50,000 – 150,000 in a single individual ant brain, and thus is expected to be sufficient for capturing most cell types in the ant brain.
Data exclusions	No data were excluded.
Replication	Four to five replicates were analyzed for each adult phenotype to ensure the reproducibility of the findings.
Randomization	Samples were allocated into different groups according to their adult phenotype identity (i.e. workers, queens, gynes or males).
Blinding	Blinding was not relevant to this study because the adult phenotype identity (i.e. workers, queens, gynes or males) of each collected sample was very clear.

Reporting for specific materials, systems and methods

We require information from authors about some types of materials, experimental systems and methods used in many studies. Here, indicate whether each material, system or method listed is relevant to your study. If you are not sure if a list item applies to your research, read the appropriate section before selecting a response.

Materials & experimental systems

n/a	Involved in the study	
☒	☐	Antibodies
☒	☐	Eukaryotic cell lines
☒	☐	Palaeontology and archaeology
☐	☒	Animals and other organisms
☒	☐	Human research participants
☒	☐	Clinical data
☒	☐	Dual use research of concern

Methods

n/a	Involved in the study	
☒	☐	ChIP-seq
☒	☐	Flow cytometry
☒	☐	MRI-based neuroimaging

Animals and other organisms

Policy information about studies involving animals; ARRIVE guidelines recommended for reporting animal research

Laboratory animals	The original colony of Monomorium pharaonis was collected in 2016 from a resident house in Mengla, Xishuangbanna, Yunnan Province, China, and split into hundreds of sub-colonies in the lab in the subsequent years. All colonies were reared at 27℃, 65% RH and a 12/12 hr light/dark cycle. The rearing of gynes and males was induced in newly split colonies where inseminated and egg-laying queens were removed, and where easily recognizable male pupae were continuously removed to prevent that newly hatching gynes became inseminated. The eclosion date of males and gynes were recorded. The queens were collected from stable, mature colonies in which they were actively laying eggs. The demographic states of the colonies were frequently surveyed, so the ages of queens could be estimated, albeit less accurately than the gynes and males. Workers were randomly collected from colonies, both inside and outside of nests, so these samples covered both young (nursing) and old (foraging) workers. At the moment of dissection, gynes were 5-10 days post-eclosion, queens were 3-6 months post-eclosion, and males were 3-14 days post-eclosion, while the age of workers was not recorded.
Wild animals	The study did not involve wild animals.
Field-collected samples	The study did not involve samples collected from the field.
Ethics oversight	No ethical approval was required, as the studied species is an ant species that is commonly found around the world and can be easily maintained in lab.

Note that full information on the approval of the study protocol must also be provided in the manuscript.
