# Supplementary figures and images for: A single-cell transcriptomic atlas tracking the neural basis of division of labour in an ant superorganism
Source: Nat Ecol Evol. 2022 Jun 16;6(8):1191–204. doi: 10.1038/s41559-022-01784-1 (PMC9349048; doi:10.1038/s41559-022-01784-1)

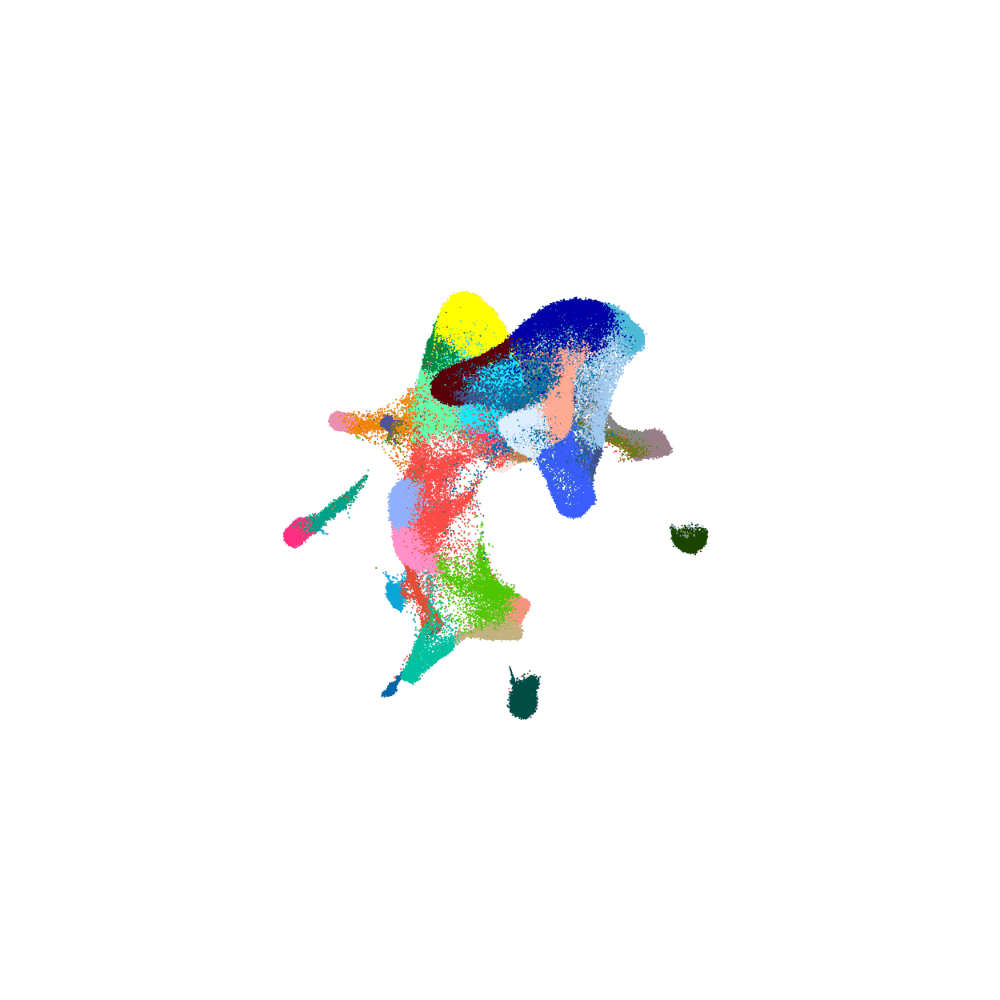

Supplement: Supplementary file 5 — A 3D view for the 43 Monomorium cell clusters. [file 41559_2022_1784_MOESM5_ESM.gif]
